# Primary Care Pharmacy Competencies of Graduates from a Community-Focused Curriculum: Self- and Co-Worker Assessments

**DOI:** 10.3390/pharmacy13050139

**Published:** 2025-10-01

**Authors:** Kritsanee Saramunee, Chakravudh Srirawatra, Pathinya Buaban, Surasak Chaiyasong, Wiraphol Phimarn

**Affiliations:** Social Pharmacy Research Unit, Faculty of Pharmacy, Mahasarakham University, Maha Sarakham 44150, Thailand

**Keywords:** competency assessment, pharmacy graduates, primary care pharmacy, home visit, community engagement

## Abstract

Primary Care Pharmacy (PCP) plays a vital role in healthcare systems. This study evaluated the competencies of pharmacy graduates from a community-focused curriculum, emphasizing their skills and personal traits. A structured questionnaire assessed four domains: general characteristics (11 items), PCP skills (16 items: 13 home visit and 3 community engagement skills), PCP personal traits (7 items), and readiness for PCP practice. Two sets of questionnaires were distributed in 2018 to recent pharmacy graduates: one for self-assessment and the other for evaluation by supervisors or co-workers. A 5-point scale (1 = least competent, 5 = most competent) was used. Co-workers gave higher scores than the graduates themselves. In home visit skills, “providing medicine advice” scored highest (4.4 ± 0.6 by graduates; 4.5 ± 0.2 by co-workers), while “performing essential physical exams” scored the lowest (3.5 ± 0.7). For co-workers, the lowest score was “working with a multidisciplinary team” (3.9 ± 0.9). Among community engagement skills, “solving health-related problems” rated highest (3.4 ± 0.7), and “identifying community health needs” rated lowest (3.2 ± 0.7). “Being friendly” and “responsibility” were top-rated personal traits by graduates and co-workers, respectively. The lowest was “coordinating with local organizations.” Graduates showed strong PCP traits and home visit skills but moderate community engagement. Community-based exposure is recommended to enhance these competencies.

## 1. Introduction

The World Health Organization’s 2018 Declaration of Astana states that primary healthcare and health services should be of high quality and safe, comprehensive, integrated, accessible, available, and affordable to everyone. This vision aims to achieve universal health coverage and sustainable goals [1]. The primary healthcare workforce, including pharmacists, plays a crucial role in promoting community health. Their responsibilities include promoting integrated health services, community engagement, multisectoral actions, and users [2]. A competent workforce is the key to the success of these ambitious goals.

Primary care pharmacy (PCP) focuses on ensuring equitable access to safe, effective, and affordable medicines and vaccines within the community. PCP also fosters medicine and vaccine literacy, encourages responsible self-care and self-management, and extends services such as home medication management in collaboration with multidisciplinary teams [3,4]. In addition, primary care pharmacists often lead community campaigns to promote rational medicine use [5].

Building on these roles, pharmacists worldwide are increasingly integrated into primary care teams, contributing to prescribing, chronic disease management, and medication review. These developments are supported by competency frameworks such as the FIP Global Competency Framework and national standards in the UK, Canada, and Australia [6,7]. In Thailand, primary care is delivered through government-organized primary care units, where pharmacists play key roles in medication management, interdisciplinary home visits, and community health promotion [8,9,10]. Although the Thai PharmD program incorporates community and public health experiential learning, systematic assessment of pharmacists’ primary care competencies remains limited.

Competency in PCP encompasses fundamental knowledge, clinical and analytical skills, and patient education, which are routinely assessed by pharmacy schools and national licensing examinations. Interprofessional competencies and skills for community development are equally important [2], as emphasized in the national pharmacy competencies of Australia [11] and Thailand [12]. However, assessing these competencies is challenging due to their subjective nature. Training pharmacy students in these intangible skills improves the quality of pharmaceutical services and helps them respond better to population needs [3]. Although pharmacy competencies are well documented in hospitals and community pharmacies [13,14], little is known about the competencies required of primary care pharmacists working directly within communities.

Community-based education (CBE) is strategically important for providing contextual learning to medical students. However, teaching guidance for CBE remains unclear [15,16]. Teaching methods, ranging from lecture-based to problem-based learning, can enhance primary care competency [17]. A previous US study demonstrated a community-based learning model for second-year pharmacy students in which instructors assigned students to observe and propose a workflow for the service area within a participating community pharmacy to increase revenue. Upon completing the activity, students self-rated that they felt prepared to develop a collaborative practice agreement project in the community pharmacy [18]. Practicing in primary care centers is another example of CBE for medical students that has been demonstrated to provide positive benefits to clinical knowledge and skills [19]. A 2-week community-based learning activity aimed at enhancing interprofessional skills among Thai medical and health promotion students was initiated. Students created health initiatives that matched community needs. This activity significantly increases collaborative competencies [20]. However, learning activities that allow pharmacy students to experience community life and engage in human lifestyles are scarce. Additionally, the skills required for home visits and community engagement have rarely been reported and remain challenging to teach.

### 1.1. A Community-Focused Pharmacy Curriculum

In Thailand, most pharmacy schools have fostered student experiences of working in the community, working with service providers (e.g., community pharmacies and primary care centers), and engaging with individuals in the community. The Faculty of Pharmacy at Mahasarakham University delivers a community-based learning activity integrated into the 6-year pharmacy curriculum that is a compulsory component for all pharmacy students. The activity was structured for second- and fifth-year students, each with specific objectives and tasks designed to enhance community engagement and home-visit skills.

#### 1.1.1. Year 2 (Junior Students)

In their second year, the students participated in community-based activities focused on two key subjects. During the first semester, in the 2-credit-course titled “Social Pharmacy” (30 h), students were assigned to visit a community located within a 10 km radius of the pharmacy faculty. Their task was to gain an overview of the community (knowing its nature) using various community-learning tools such as geographical maps, family trees, community calendars, and respected person biographies. A group of 10 to 12 students was assigned to explore the nature of the community. Four hours were reserved for the activity.

In the second semester of the 2-credit-course titled “Public Health Pharmacy” (30 h), the students continued to engage with the same community. A group of 3 to 5 students was assigned to visit a host family at home where they were expected to approach the host appropriately, perform basic history-taking, and practice obtaining basic health data such as Body Mass Index (BMI), smoking habits, and medication use [21]. Four hours were reserved for this activity.

For both activities, the social and administrative pharmacy teaching teams initially coordinated with the community and facilitated students to complete assignments.

#### 1.1.2. Year 5 (Senior Students)

In their fifth year, the students undertook more intensive community-based activities. This was part of the 2-credit-course titled “Health Promotion” that consisted of 30 h plus 15 h allocated from other courses. The students stayed with their host families who agreed to accommodate them for seven days (six nights). During this period, students immersed themselves in the daily lives of their host families and explored and analyzed the health situation of the community. Gathering social and health information led to the development and implementation of health promotion campaigns and rational medicine use initiatives within the community. Community services provided during this camp included health screening, health education, medication advice, and home visits [22].

Social and administrative pharmacy teaching teams were initially coordinated with the community. However, students must take the lead in organizing this community service. They designed the organizational structure of the team and assigned team members specific responsibilities such as coordinating with community leaders, financial management, catering services, and academic services. The Faculty of Pharmacy at Mahasara-kham University began this community-based learning activity in 2005 and has continued to use it. This activity has been modified periodically.

These compulsory activities were designed to enhance student skills in conducting home visits and engaging with the community. However, this competency has not been appropriately assessed. Consequently, this study aimed to assess the competencies of pharmacy graduates in PCPs, with a specific focus on their skills in delivering home visits and engaging with the community. The purpose of this study was to introduce a distinguished model of community-based learning and demonstrate its impact on PCP competencies. We hypothesized that pharmacy graduates would rate their PCP competencies as good. These areas have been neglected in previous research. This study seeks to address this gap by providing valuable insights into pharmacy education and practice.

## 2. Materials and Methods

### 2.1. Study Design

A cross-sectional survey was conducted in 2018 among pharmacy graduates of the Faculty of Pharmacy at Mahsarakham University who used a community-focused pharmacy curriculum. All students were involved in the community-based learning activities mentioned above. The study population comprised pharmacy graduates of the class 2015–2017, as they graduated most recently during the data collection period. The target sample size was calculated from the estimated population proportion using Cochran’s formula [12]: *n* = [*z*^2^ × *p* × (1 − *p*)]/*e*^2^. Replacing these parameters in the formula, *z* is the *z* value at the significance level replaced by 1.64 for a confidence level of 90%, *p* is the population proportion replaced by 0.50 to maximize the sample size, and *e* indicates acceptable sampling error and was replaced by 0.05. This yielded a target sample size of 269 per group, graduates and co-workers. However, the alumni repository contained only 250 graduate contact addresses. Questionnaires were therefore distributed to these 250 workplaces, with one graduate and one co-worker invited to participate at each site.

### 2.2. PCP Competencies

PCP competencies were reviewed and extracted from the previous literature [8,9,23,24] while focusing on the skills pharmacists should possess to provide home visits and work with the community. We pre-identified a set of PCP competencies encompassing three domains, including clinical skills for home visits, engagement with the community, and personal traits. Some clinical pharmacy skills are essential for home visits such as providing medicine and health advice, communicating with patients and multidisciplinary teams, and medication review and management [8,23]. Engaging with the community is a special skill for PCP in dealing with community and public health [8,23,24]. Personal characteristics such as interpersonal skills, responsibility, and accountability for assigned jobs are also essential [9]. A questionnaire was drafted and sent for a validity assessment.

### 2.3. Questionnaire Validation

Three experienced primary care pharmacists assessed the content validity. The first draft was reviewed and assessed for content, and comments were added. We have revised the first draft of the questionnaire based on this feedback. The second version of the questionnaire was sent to ten primary care pharmacists who had worked closely with the community for at least one year to re-assess the soundness of the competency statements and test the questionnaire. We have revised the second draft of the questionnaire based on this feedback. The third version of the questionnaire was sent to 14 pharmacy alumni, and seven co-workers participated in this pilot study to ensure the feasibility of collecting data and performing reliability tests. Amendments have been made to this version; however, none have affected the content of the key questions.

The questionnaire was composed of four parts: Part I general characteristics—11 items composed of close-ended questions; Part II PCP skills—16 items composed of 13 home visit skills and three community engagement skills (five-point Likert scale: 1 = least competent; 5 = most competent); Part III PCP personal traits—seven items (five-point Likert scale: 1 = least competent; 5 = most competent); Part IV readiness for delivering a PCP measured by a visual analog scale of a 10 cm line (0 = not ready; 10 = most ready). An open-ended question was included to collect graduates’ comments on the learning activity. Domain scores are expressed as means with standard deviations, preserving the 5-point Likert scale and facilitating cross-domain comparisons.

The questionnaire was validated by the validscale command in STATA version 15 [25]. The correlations between competency items and domains were good with values ranging between 0.524–0.886. All items exhibited good convergence with the scores on their own dimensions. Ninety-five percent of the items within the same domain did not correlate with the scores of the other domains, thus indicating good divergent validity. Reliability was tested with the questions used in Parts II and III and confirmed internal consistency as evidenced by Cronbach’s alpha coefficients of 0.935, 0.938, 0.966, and 0.968 for home-visit skills, community engagement skills, personal traits, and overall, respectively.

### 2.4. Data Collection

A package containing an invitation letter, two questionnaires, information sheets, consent forms, and a prepaid envelope was posted at the pharmacy graduates’ workplaces. A pharmacist who was our pharmacy graduate and one of their co-workers were invited to respond to this survey. Data were collected between March and April 2018. Pharmacy graduates who did not complete the survey within one month were followed up by telephone.

### 2.5. Statistical Analysis

Frequencies and percentages are reported as appropriate. The mean score for each competency item was calculated. The Kruskal–Wallis test was used in the subgroup analysis to confirm the associations between general characteristics and self-assessment competency scores. The Mann–Whitney U test was used to identify differences between self-assessments and co-worker assessments. Cohen’s *d* was computed as a standardized measure of effect size for differences between self- and co-worker assessments. Comments from the open-ended question were reviewed and grouped to provide a descriptive summary.

### 2.6. Ethical Consideration

This study was conducted in accordance with the Good Clinical Practice Guidelines for Research. This study was reviewed and approved by the institutional review board of Mahasarakham University (approval code: 019/2560, dated 1 February 2018). Only key demographic data were collected, and personal identities were not recorded to ensure participant anonymity and confidentiality.

## 3. Results

### 3.1. Survey Respondents

Of the 250 distributed questionnaires, 103 were returned, yielding response rates of 41.2% (graduates) and 30.8% (co-workers). Pharmacy graduates were primarily female (78.6%), graduated in 2017 (45.6%), worked in public hospitals (82.5%), and were involved in medicine dispensing and managing medicine supplies in primary care centers (42.3%). More than one-third of the graduates were already responsible for PCP (38.2%). Co-workers primarily completed the 6-year PharmD curriculum (53.2%) and worked in public hospitals (89.6%). More than half of the cases involved PCP (Table 1).

### 3.2. Primary Care Pharmacy Competency

**Figure 1:**
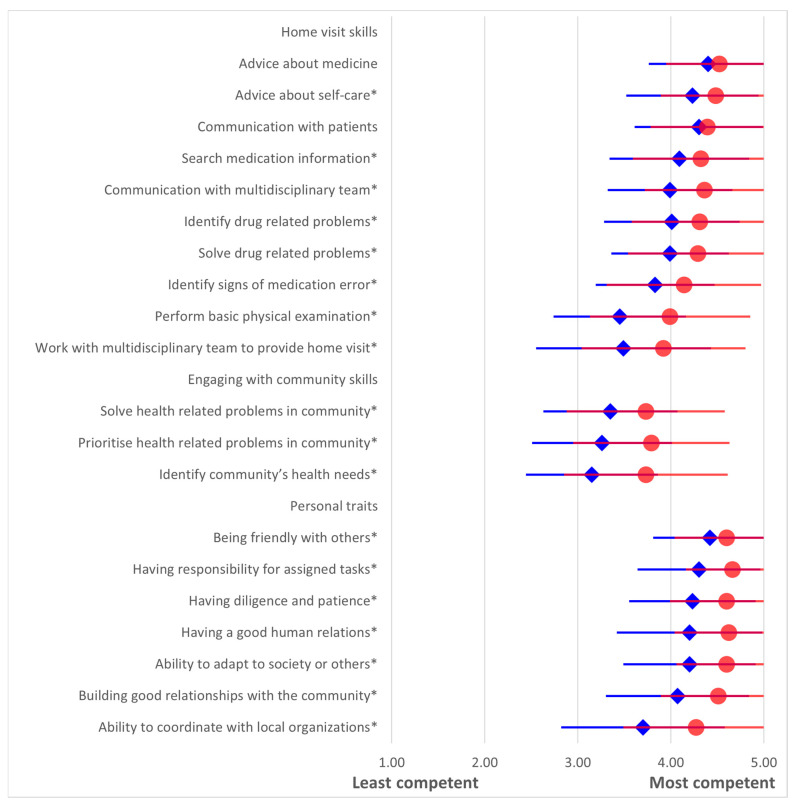
Primary care pharmacy competencies rated by pharmacy graduates (self-assessment, blue diamonds) and their co-workers (red circles). Data were collected in 2018 from graduates of classes 2015–2017, Mahasarakham University. Scores represent means with standard deviations on a 5-point Likert scale (1 = least competent, 5 = most competent). Asterisks indicate statistically significant differences between groups (*p* < 0.05, Mann–Whitney U test). Effect sizes (Cohen’s *d*) for significant items are reported in Supplementary Table S2.

The PCP competencies are illustrated in Figure 1. Overall, the scores for most items were higher when rated by co-workers compared to the self-assessments (Mann–Whitney U test, *p* < 0.05). The competency scores for home-visit skills and personal characteristics were greater than 4.0 points, thus indicating good competencies among respondents. However, there were also scores of between 3.0–4.0 points that were indicative of moderate engagement in community skills.

Among home visit skills, providing medicine advice received the highest ratings (4.4 ± 0.6 from graduates and 4.5 ± 0.2 from co-workers). Graduates rated their ability to perform basic physical examinations lowest (3.5 ± 0.7), while co-workers rated working with a multidisciplinary team for home visits lowest (3.9 ± 0.9). Most of home visit skills were rated above 4.00 points, with the exceptions of “performing basic physical examination” and “working with multidisciplinary team to provide home visit” that were below 4.0 points. Communication with patients was rated similarly between the self- and co-worker assessments (*p* = 0.49), thus indicating a common agreement on this item from the two perspectives.

In terms of engaging with the community, graduates rated “solving health-related problems in the community” highest (3.4 ± 0.7) and “identifying the community’s health needs” lowest (3.2 ± 0.7). Co-workers rated all three community engagement skills similarly, and they provided higher scores than did graduates.

Regarding personal traits, “being friendly with others” was rated highest by graduates (4.4 ± 0.6), while co-workers rated “having responsibility for assigned tasks” highest (4.7 ± 0.5). The ability to coordinate with local organizations was rated lowest by both groups (3.7 ± 0.9 by graduates and 4.3 ± 0.8 by co-workers). All personal characteristics were rated above 4.0 points, although the ability to coordinate with the local organization was lower than that of other characteristics. Co-workers rated almost 5.0 points for most items in this domain, with the exception of “ability to coordinate with the local organization” (4.3 ± 0.8).

Subgroup analysis using the Kruskal–Wallis test demonstrated that several self-assessment scores were dominated by the general characteristics of pharmacy graduates. (Table 2). Pharmacy graduates involved in home visits are likely to be more competent in regard to advising on medicine (*p* = 0.018). Those working in community pharmacies were likely to be more competent in providing basic physical examinations (*p* = 0.011) and identifying drug-related problems (*p* = 0.035). Males were likely to rate higher scores than females for competency to “solving drug-related problems” (*p* = 0.048) and “working with a multidisciplinary team to provide home visits” (*p* = 0.043). Pharmacy graduates already involved in all PCP tasks were likely to be more competent at working with multidisciplinary teams to provide home visits (*p* < 0.05). Graduates working in community pharmacies are likely to be more competent at building good relationships with the community (*p* = 0.046).

### 3.3. Readiness for Delivering Primary Care Pharmacy

**Figure 2:**
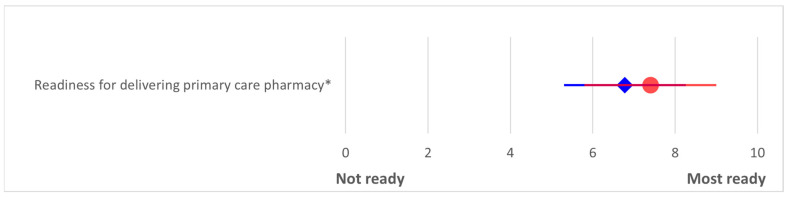
Readiness to deliver primary care pharmacy rated by pharmacy graduates (self-assessment, blue diamonds) and their co-workers (red circles). Data were collected in 2018 from graduates of classes 2015–2017, Mahasarakham University. Scores represent means with standard deviations on a 5-point Likert scale. Asterisks denote statistically significant differences between groups (*p* < 0.05, Mann–Whitney U test).

Readiness for delivering PCP was rated 6.8 ± 1.5 and 7.4 ± 1.6 by pharmacy graduates and co-workers, respectively. Again, the score rated by co-workers was significantly higher than was the graduate self-assessment (Mann–Whitney U test, *p* = 0.002). (Figure 2). Almost all pharmacy graduates (102, 99.0%) agreed that community-based activities embedded in the pharmacy curriculum helped enhance their PCP competencies. Examples of comments from the pharmacy graduates are as follows:

“This is a strength of the pharmacy curriculum here because it helps us see true problems in the community.”

“This kind of activity should be continued because it helps students become familiar with the community lifestyle.”

“It would be better for students to work with the primary care center to search and identify patients with drug-related problems.”

## 4. Discussion

This study demonstrated the PCP competencies gained through integrating community-based learning activities into the pharmacy curriculum. Previous studies have assessed pharmacy competencies in hospitals and community pharmacies [13,14], but this is the first endeavor to assess pharmacy competencies specifically for working directly with the community. Healthcare provider competency encompasses knowledge, skills, abilities, and traits, which can be developed through education, training, and practice. These competencies are assessed using various methods (e.g., written tests, case simulations, and supervisory performance appraisals) and by different assessors (self, peer, supervisor, expert) [26]. Because self-assessment may be subject to positive bias [27], we included co-worker assessments to balance the findings.

Three main skills were identified, including home visits, community engagement, and personal traits. Although home visit skills and personal traits appear similar to those essential for working in other pharmacy settings, their necessity has been confirmed by the literature [8,9,23,24] and our experts. Engaging with the community is considered a specific skill by primary care pharmacists.

Unlike previous studies [16,18], this learning model takes students out of their comfort zones and immerses them in a real community culture and lifestyle. Nearly all respondents (99.0%) agreed that this experience enhanced their PCP competencies, ultimately leaving them feeling prepared to deliver PCP services (average readiness score = 6.8 ± 1.5, out of 10). While two previous studies used a similar model with a specific class [15,20], one observed that it enhanced the professional development of final-year physiotherapy students [15], and the other demonstrated a significant increase in collaborative competencies among second-year medical and health promotion students [20]. A distinctive feature of the learning model employed at Mahasarakham University in Thailand is its application across both junior and senior students, with the level of challenge increasing in tandem with student professional maturity.

Competency scores for home visit skills and personal characteristics were consistently above 4.00 points, thus indicating competence. However, the scores related to community engagement skills were noticeably lower. In particular, graduates rated their “ability to coordinate with local organizations” that is a key personal trait at 3.7 ± 0.9. This gap in community engagement skills could be attributed to the specific responsibilities assigned to students. Often, only student leaders have the opportunity to communicate with the community or handle unexpected events. As a result, these skills may have developed unevenly among all students.

The pharmacy graduate respondents were reluctant to engage with the community by solving health-related problems (3.4 ± 0.7), identifying community health needs (3.2 ± 0.7), or working with local organizations (3.7 ± 0.9). As pharmacists are experts in medicine, these skills may seem indirectly related to their roles. The pharmacy curriculum primarily focuses on pharmacy disciplines and community-based activities, although these are innovative and constitute only a small portion of the curriculum. Therefore, pharmacists involved in primary care may require postgraduate training to develop these skills further.

A considerable proportion (38.2%) of pharmacy graduates possessed responsibilities related to PCP. Although we did not obtain comparative data from other schools, it is important to note that there are currently no specific requirements for assigning PCP roles to novice pharmacists. Nevertheless, implementing community-based training may be necessary to better prepare graduates for these roles.

This study illustrates that pharmacy graduates possess strong home-visit skills, particularly clinical skills and communication (average score ≥ 4.0). However, their skills in regard to performing basic physical examinations (3.5 ± 0.7) and working with multidisciplinary teams (3.5 ± 0.9) were moderate. A previous study reported that Canadian pharmacists were confident when monitoring vital signs but less confident when performing palpation, percussion, and auscultation [28]. Primary care pharmacists are encouraged to perform simple physical examinations such as monitoring vital signs, assessing pitting edema, and checking for diabetic peripheral neuropathy. These skills are taught in pharmacy schools, as they are essential for providing appropriate pharmacotherapy and communicating patient status to other health professionals during home visits.

Previous studies have reported the positive effects of CBE activities on teamwork and lifelong learning skills [29,30]. One quasi-experiment demonstrated a positive effect of CBE activity on teamwork skills. Egyptian nursing students who delivered a 3-week-rotation of health education via posters, brochures, booklets, and banners in rural communities increased their teamwork skills significantly after the implementation of such activities [29]. Students at the University of Southern California spent two days of community immersion exploring, observing, and examining the societal factors that impact the community. This activity appears to enhance student teamwork and lifelong learning skills [30]. However, this study did not assess the impact of PCP skills on other soft skills, and this may be a question for future research.

Interestingly, the subgroup analysis revealed that graduates working in community pharmacies were more competent when performing basic physical examinations (*p* = 0.011). This may be due to the observation these skills are part of the daily practice in community pharmacies. Working with multidisciplinary teams for home visits is crucial in primary care. Subgroup analysis demonstrated that graduates involved in all PCP tasks were more competent in this area (*p* < 0.05). Previous evidence suggests that having other health professionals in a team can enhance the success of PCP services [9], thus indicating that working with other professionals is essential, and students must be confident in this regard. The pharmacy graduates expressed positive feedback on the community-based learning model. These findings underscore the need for experiential learning activities for pharmacy students.

### 4.1. Implications for Pharmacy Education

Primary care is becoming increasingly important, as it is key to strengthening health for all nations, thus highlighting the necessity for pharmacists to have these skills. While other pharmacy schools in Thailand provide community experiences for their students, learning activities and their intensity vary depending on resources and readiness. However, this model has rarely been applied in other countries. Our study suggests that students need to gain experience in the actual community culture. Therefore, pharmacy schools should provide students with this experience, particularly in countries where pharmacists work closely with the community members.

Implementing community-based learning activities requires a strong and close connection between pharmacy faculty and the community. The administrative board should identify the right place and establish a memorandum of understanding to maintain the activity. This activity can be a part of a community service that is frequently cited as a core function of higher education institutions [31].

A longitudinal study is required to determine the long-term impact of community-based learning on pharmacy students. Pharmacy schools may consider using this PCP competency assessment questionnaire, as it exhibited good validity and reliability. The assessment should be implemented upon completion of student activity to self-monitor competency during training. Furthermore, specific PCP skills and competencies may be sought from student perspectives, and discussing this issue with them upon completion of the activity would be valuable.

### 4.2. Strengths and Limitations

This study provides an example of using a real community as a practice-based learning environment for pharmacy students and demonstrates the feasibility of integrating community-based education into the pharmacy curriculum. Competency was assessed using both self-assessments and co-worker assessments to minimize bias from self-assessment overestimation. Interestingly, self-assessment scores were lower in all domains, indicating either understated self-evaluation or lower confidence. In a non-experimental study, the reported competency scores may have been influenced by other factors such as extracurricular activities that were not observed in this study. Designing an experimental study with a control group was difficult, as this community-based activity was compulsory for all students. Future research should consider using hospital pharmacists as controls to confirm the effects of this activity on PCP competency. Although the calculated target sample size was 269 per group, only 130 graduate and 77 co-worker responses were obtained. This smaller sample size may reduce the precision of estimates and generalizability. The power of the test was as high as 99.1% for co-worker samples but only 24.7% for pharmacy graduate samples (*n* = 103). This was due to the greater effect size among the pharmacy graduate samples, resulting in a small chance of detecting a true effect. This phenomenon limits generalizability and test power, and therefore, interpretations should be made cautiously. The questionnaire was not co-developed with external social scientists, which may have enriched its design. Nonetheless, it was developed under the guidance of social pharmacy expertise within the team and underwent iterative validation to ensure content validity and reliability.

## 5. Conclusions

Pharmacy graduates exposed to a community-focused pharmacy curriculum demonstrated high competence in regard to providing home visits and exhibited strong personal characteristics, whereas their community engagement skills were moderate. Experience with the community culture is necessary to enhance PCP competencies, and community-based activities are recommended for pharmacy schools. A longitudinal study is required to determine the long-term impact of community-based learning on pharmacy students. These findings are valuable for improving community-based activities in pharmacy curricula.

## Figures and Tables

**Table 1:** General characteristics of pharmacy graduates (classes of 2015–2017, Mahasarakham University) and their co-workers who participated in the study conducted in 2018.

	Graduates (*n* = 103)	Co-Workers (*n* = 77)
	*n* (%)	*n* (%)
Gender		
Male	22 (21.4)	24 (31.2)
Female	81 (78.6)	53 (68.8)
Age (yrs)		
20–29	101 (99.0)	37 (48.1)
30–39	1 (0.9)	29 (37.7)
40–49	-	10 (12.9)
>50	-	1 (1.3)
Graduation year		
Class 2015	23 (22.3)	-
Class 2016	33 (32.0)	-
Class 2017	47 (45.6)	-
Co-workers highest education		
5-year pharmacy curriculum	-	29 (37.7)
6-year PharmD curriculum	-	41 (53.2)
Master’s degree	-	6 (7.8)
Current workplace ^a^		
Public hospital	85 (82.5)	69 (89.6)
Private hospital	-	1 (1.3)
Community pharmacy	17 (16.5)	14 (18.2)
Pharmaceutical company	1 (0.9)	-
Responsible for primary care pharmacy	39 (38.2)	45 (59.2)
Roles of primary care pharmacy ^a^		
Consumer health protection in community	11 (15.5)	22 (33.9)
Home visit	17 (23.9)	23 (35.4)
Medicine dispensing and managing medicine supply in primary care center	30 (42.3)	36 (55.4)

Remark: ^a^ Values may total more than 100% because responses were not mutually exclusive.

**Table 2:** Associations between graduates’ general characteristics and their self-assessed primary care pharmacy competency scores (graduates from classes 2015–2017, Mahasarakham University, surveyed in 2018).

Competencies	Mean	SD	*p*
**Home visit skills**			
*Advice about medicine*			
Involve in home visit (*n* = 17)	4.7	0.5	0.018
Not involve in home visit (*n* = 54)	4.3	0.6	
*Perform basic physical examination*			
Work in community pharmacy (*n* = 17)	3.8	0.1	0.011
Not work in community pharmacy (*n* = 86)	3.4	0.7	
*Identify drug related problems*			
Work in community pharmacy (*n* = 17)	4.4	0.6	0.035
Not work in community pharmacy (*n* = 86)	3.9	0.7	
*Solve drug related problems*			
Male (*n* = 22)	4.2	0.7	0.048
Female (*n* = 81)	3.9	0.6	
*Work with multidisciplinary team to provide home visit*			
Male (*n* = 22)	3.9	0.9	0.043
Female (*n* = 81)	3.4	0.9	
Responsible for primary care pharmacy (*n* = 39)	3.8	0.9	0.004
Not responsible for primary care pharmacy (*n* = 63)	3.3	0.9	
Involve in consumer health protection (*n* = 11)	4.2	0.9	0.031
Not involve in consumer health protection (*n* = 60)	3.5	0.9	
Involve in home visit (*n* = 17)	4.2	0.8	0.001
Not involve in home visit (*n* = 54)	3.4	0.9	
Involve in medicine dispensing and managing medicine supply in primary care (*n* = 30)	3.9	0.9	0.026
Not involve in medicine dispensing and managing medicine supply in primary care (*n* = 41)	3.3	0.9	
**Personal characters**			
*Building good relationships with the community*			
Work in community pharmacy (*n* = 17)	4.4	0.6	0.046
Not work in community pharmacy (*n* = 86)	4.0	0.8	

Remark: *p* was from Kruskal–Wallis test.

## Data Availability

The data presented in this study are available on request from the corresponding author due to ethical reasons.

## Supplementary Materials

The following supporting information can be downloaded at: https://www.mdpi.com/article/10.3390/pharmacy13050139/s1, Table S1: Bilingual questionnaire; Table S2: Cohen’s *d* effect sizes for self- and co-worker assessments of primary care pharmacy competencies.

## Abbreviations

The following abbreviations are used in this manuscript:

PCP	Primary Care Pharmacy
CBE	Community-based education
BMI	Body Mass Index
