# Supplementary material for: Primary Care Pharmacy Competencies of Graduates from a Community-Focused Curriculum: Self- and Co-Worker Assessments
Source: Pharmacy (Basel). 2025 Oct 1;13(5):139. doi: 10.3390/pharmacy13050139 (PMC12567282; doi:10.3390/pharmacy13050139)
Supplement: Supplementary file 1 [file pharmacy-13-00139-s001.zip › pharmacy-3823876-supplementary.pdf]

Table S1 Bilingual questionnaire

ตอนที่ 1 ข้อมูลทั่วไปของผู้ตอบแบบสอบถาม

Part 1 General information of respondents

1. เพศ / Gender

□ ชาย / Male □ หญิง / Female

2. อายุ / Age

□ 20–29 ปี / 20–29 years

□ 30–39 ปี / 30–39 years

□ 40–49 ปี / 40–49 years

□ 50 ปีขึ้นไป / ≥50 years

3. ปีที่จบการศึกษาจากคณะเภสัชศาสตร์ / Year of graduation from Faculty of Pharmacy

□ พ.ศ. 2558 (รุ่นที่ 10) / 2015 (Cohort 10)

□ พ.ศ. 2559 (รุ่นที่ 11) / 2016 (Cohort 11)

□ พ.ศ. 2560 (รุ่นที่ 12) / 2017 (Cohort 12)

□ อื่น ๆ ระบุ .... / Others (specify) …………

4. สถานที่ทำงานปัจจุบัน (เลือกตอบได้มากกว่า 1 ข้อ) / Current workplace (choose more than one if applicable)

□ โรงพยาบาลในหน่วยงานภาครัฐ / Government hospital

□ สำนักงานสาธารณสุขจังหวัด / Provincial public health office

□ โรงพยาบาลเอกชน / Private hospital

□ ร้านยา / Community pharmacy

□ บริษัทยา / Pharmaceutical company

□ อื่น ๆ .... / Others …………

5. ท่านได้รับผิดชอบงานเภสัชกรรมปฐมภูมิหรือไม่ / Are you responsible for primary care pharmacy services?

□ ได้ / Yes □ ไม่ได้ / No

6. โปรดระบุขอบเขตงานเภสัชกรรมปฐมภูมิที่ท่านรับผิดชอบ (เลือกตอบได้มากกว่า 1 ข้อ)

Please specify the scope of primary care pharmacy services you are responsible for (choose more than one if applicable)

□ งานคุ้มครองผู้บริโภคในชุมชน / Consumer protection in the community

□ งานเยี่ยมบ้าน / Home visits

Permission is required before use

□ งานดูแลส่งมอบยาและการใช้ยาใน รพ.สต. / Medicine supply and use in primary care unit
□ อื่น ๆ ระบุ ………… / Others …………

ตอนที่ 2 ทักษะที่ใช้สำหรับการบริการเภสัชกรรมปฐมภูมิ

Part 2 Skills for Primary Care Pharmacy Services

กรุณาประเมินระดับความสามารถของท่าน (1 = น้อยที่สุด, 5 = มากที่สุด)

Please rate your competency (1 = lowest, 5 = highest)

	**ระดับความสามารถของท่าน** Competency level				
	**มากที่สุด** → **น้อยที่สุด** Highest → Lowest				
	5	4	3	2	1
**การเยี่ยมบ้าน** / Home visit					
การให้คำแนะนำการใช้ยา Advice about medicine	☐	☐	☐	☐	☐
การให้คำแนะนำในการปฏิบัติตัวแก่ผู้ป่วย Advice about self-care	☐	☐	☐	☐	☐
การสื่อสารกับผู้ป่วย Communication with patients	☐	☐	☐	☐	☐
การสื่อสารกับสหวิชาชีพ Communication with multidisciplinary team	☐	☐	☐	☐	☐
การตรวจร่างกายเบื้องต้นเพื่อค้นหาปัญหาด้านยา Perform basic physical examination	☐	☐	☐	☐	☐
การสังเกตอาการผิดปกติของผู้ป่วยที่เกี่ยวเนื่องจากการใช้ยา Identify signs of medication error	☐	☐	☐	☐	☐
การสืบค้นข้อมูลด้านยา Search medication information	☐	☐	☐	☐	☐
ในการค้นหาปัญหาด้านยา Identify drug related problems	☐	☐	☐	☐	☐
ในการแก้ไขปัญหาด้านยา Solve drug related problems	☐	☐	☐	☐	☐
การเยี่ยมบ้านร่วมกับทีมสหสาขาวิชาชีพ Work with multidisciplinary team to provide home visit	☐	☐	☐	☐	☐
**การสร้างความผูกพันกับชุมน** / Community engagement					

	ระดับความสามารถของท่าน Competency level				
	มากที่สุด ⟶ น้อยที่สุด Highest Lowest				
	5	4	3	2	1
การจัดการปัญหาที่เกี่ยวกับสุขภาพในชุมชน<br>Solve health related problems in community	☐	☐	☐	☐	☐
การจัดลำดับความสำคัญปัญหาของชุมชน<br>Prioritize health related problems in community	☐	☐	☐	☐	☐
การประเมินความต้องการด้านสุขภาพของชุมชน<br>Identify community's health needs	☐	☐	☐	☐	☐

ตอนที่ 3 คุณลักษณะส่วนบุคคลที่เหมาะสมสำหรับเภสัชกรปฐมภูมิ

Part 3 Personal Traits for Primary Care Pharmacy Services

โปรดประเมินระดับคุณลักษณะของท่าน (1 = น้อยที่สุด, 5 = มากที่สุด)

Please rate your personal traits (1 = lowest, 5 = highest)

**คุณลักษณะส่วนบุคคลที่เหมาะสมสำหรับเภสัชกรปฐมภูมิ**	**ระดับคุณลักษณะของท่าน** Personal trait level				
	มากที่สุด ⟶ น้อยที่สุด Highest Lowest				
	5	4	3	2	1
มนุษย์สัมพันธ์ที่ดี<br>Having a good human relation	☐	☐	☐	☐	☐
สามารถปรับตัวเข้ากับสังคมหรือผู้อื่นได้<br>Ability to adapt to society or others	☐	☐	☐	☐	☐
การสร้างความสัมพันธ์ที่ดีกับชุมชน<br>Building good relationships with the community	☐	☐	☐	☐	☐
ความรับผิดชอบในงานที่ได้รับมอบหมาย<br>Having responsibility for assigned tasks	☐	☐	☐	☐	☐
ความขยันและอดทน<br>Having diligence and patience	☐	☐	☐	☐	☐
มีความเป็นกันเองกับผู้อื่น<br>Being friendly with others	☐	☐	☐	☐	☐
มีความสามารถในการติดต่อประสานงานระหว่างองค์กรท้องถิ่น<br>Ability to coordinate with local organizations	☐	☐	☐	☐	☐

ท่านคิดว่าท่านมีความพร้อมต่อการทำงานเภสัชกรรมปฐมภูมิอยู่ในระดับใด

How ready do you feel to deliver PCP services?

โปรดทำเครื่องหมาย × ลงในช่วงระดับที่ตรงกับระดับความคิดเห็นของท่านมากที่สุด

Mark × on the scale that best match your opinion

(0 = ไม่มีเลย / None, 10 = มากที่สุด / Highest)

ไม่มีเลย None

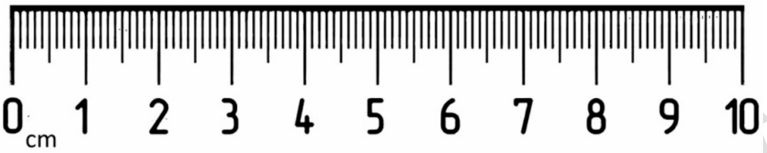

มากที่สุด Highest

ท่านคิดว่ากิจกรรมที่ใช้ชุมชนเป็นฐานในการเรียนรู้ (Community based program) ที่คณะจัดให้ช่วยส่งเสริมความสามารถการทำงานด้านเภสัชกรรมปฐมภูมิหรือไม่

Do you think community-based learning activities organized by the faculty enhanced PCP competencies?

❒ ช่วย / Yes ❒ ไม่ช่วย / No

ท่านคิดว่ากิจกรรมที่ใช้ชุมชนเป็นฐานในการเรียนรู้ (Community based program) ที่คณะจัดให้ควรมีการปรับปรุงแก้ไขหรือเพิ่มเติมในเรื่องใดบ้าง

What improvements or additions should be made to these activities?

หากท่านมีความคิดเห็นอื่น ๆ ที่มีต่อหลักสูตรเภสัชศาสตร์บัณฑิต สาขาวิชาการบริบาลทางเภสัชกรรมของคณะเภสัชศาสตร์มหาวิทยาลัยมหาสารคาม ในการเพิ่มศักยภาพบัณฑิตด้านเภสัชกรรมปฐมภูมิ

Any other comments on the PharmD curriculum?

Permission is required befo

Table S2. Cohen's *d* effect sizes for self- and co-worker assessments of primary care pharmacy competencies.

	Self-assessment by graduates (n=103)		Co-worker's assessment (n=77)		*p* value	Cohen's *d*
	Mean	SD	Mean	SD		
**Home visit skills**						
Advice about medicine	4.40	0.64	4.52	0.57	0.23	0.20
Advice about self-care*	4.23	0.71	4.48	0.59	<0.05	0.38
Communication with patients	4.30	0.69	4.39	0.61	0.49	0.14
Search medication information*	4.09	0.75	4.32	0.73	<0.05	0.31
Communication with multidisciplinary team*	3.99	0.67	4.36	0.64	<0.05	0.56
Identify drug related problems*	4.01	0.73	4.31	0.73	<0.05	0.41
Solve drug related problems*	3.99	0.63	4.29	0.75	<0.05	0.44
Identify signs of medication error*	3.83	0.64	4.14	0.83	<0.05	0.43
Perform basic physical examination*	3.45	0.71	3.99	0.86	<0.05	0.69
Work with multidisciplinary team to provide home visit*	3.49	0.94	3.92	0.88	<0.05	0.47
**Engaging with community skills**						
Solve health related problems in community*	3.35	0.72	3.73	0.85	<0.05	0.49
Prioritize health related problems in community*	3.26	0.75	3.79	0.84	<0.05	0.67
Identify community's health needs*	3.15	0.71	3.73	0.88	<0.05	0.74
**Personal traits**						
Being friendly with others*	4.42	0.61	4.60	0.56	<0.05	0.31
Having responsibility for assigned tasks*	4.30	0.66	4.66	0.50	<0.05	0.60
Having diligence and patience*	4.23	0.68	4.60	0.61	<0.05	0.57
Having a good human relations*	4.20	0.78	4.62	0.58	<0.05	0.60
Ability to adapt to society or others*	4.20	0.71	4.60	0.54	<0.05	0.62
Building good relationships with the community*	4.07	0.77	4.51	0.62	<0.05	0.62
Ability to coordinate with local organizations*	3.70	0.88	4.27	0.78	<0.05	0.68

Remark: SD = standard deviation

Permission is required before use
